# Arabinogalactan Proteins in Pollen–Pistil Interactions: Molecular Regulators of Reproductive Barriers and Compatibility

**DOI:** 10.3390/ijms27198736

**Published:** 2026-09-30

**Authors:** Oksana Alekseevna Muratova, Alexei Nikolaevich Natyrov, Tatiana Petrovna Molchanova, Larisa Ivanovna Fedoreyeva, Sergey Alekseevich Bursakov, Ekaterina Vladimirovna Zakharova

**Affiliations:** All-Russia Research Institute of Agricultural Biotechnology, 127550 Moscow, Russia; muratova.biology@gmail.com (O.A.M.); lexmitht@mail.ru (A.N.N.); tatihan@gmail.com (T.P.M.); fedlara@inbox.ru (L.I.F.); sergeymoscu@gmail.com (S.A.B.)

**Keywords:** arabinogalactan proteins, reproductive hybridization barriers, self-incompatibility, pollen, pollen tube, pollen–pistil system, intercellular interactions

## Abstract

Arabinogalactan proteins (AGPs) are a family of highly glycosylated proteins that play an important role in the reproductive process of flowering plants. This review summarizes current knowledge of arabinogalactan proteins at various stages of male and female gametophyte development and in the regulation of their interactions—from pollen grain contact with the stigma, through pollen tube (PT) germination and its guided growth toward the embryo sac, to sperm cell delivery to the egg cell. Particular attention is given to the involvement of AGPs in pollen–pistil recognition, pollen tube growth and guidance, self-incompatibility, and reproductive barriers associated with distant hybridization. By integrating recent findings, we highlight the diverse structural and functional roles of AGPs in reproductive processes and discuss current limitations in understanding their molecular mechanisms. The review also identifies key open questions and promising directions for future research on AGP-mediated regulation of reproductive interactions in angiosperms.

## 1. Introduction

The process of sexual reproduction in angiosperms represents a complex and highly organized sequence of events, involving the interaction of the male gametophyte (pollen grain (PG) and pollen tube (PT)) with the structures of the pistil (stigma, style, ovary) and the fusion of gametes within the female gametophyte (embryo sac) [1]. Intercellular communication in the pollen–pistil system is a key link in ensuring successful fertilization. Through this interaction, recognition of compatible pollen occurs, initiation of its germination takes place, directed growth of the PT toward the ovule is achieved, and delivery of sperm cells to the egg cell is accomplished [2,3]. Upon deposition of the PG onto the stigma surface, primary recognition occurs along with the activation of signals required for adhesion, hydration, and PT germination. Subsequently, the PT grows through the transmitting tissues of the style toward the embryo sac, receiving guidance signals (signaling peptides, lipids, sugars, and others) [3], where double fertilization occurs in angiosperms. Disruptions in these intercellular interactions lead to the emergence of various reproductive hybridization barriers (failure of PG germination, arrest of PT growth at different stages of germination and growth) [4,5]. Thus, the interaction between pollen and pistil represents a fundamental biological system that ensures selectivity and precision of fertilization in flowering plants. Arabinogalactan proteins (AGPs) are important components of this biological system [6].

AGPs have been found throughout the plant kingdom, from bryophytes to angiosperms and even in algae, indicating their evolutionary significance and, apparently, are nearly ubiquitous in plants [6,7]. Currently, AGPs are regarded as multifunctional components of the cell wall and extracellular matrix, involved in the regulation of various plant growth and developmental processes. Their roles have been demonstrated in cell proliferation and differentiation, cell wall formation and modification, cellular signal transduction, embryogenesis, programmed cell death, organ abscission, as well as in reproductive processes, including microsporogenesis, pollen development, PT growth, and the interaction between the female and male gametophytes [8,9,10,11,12,13,14].

Furthermore, a small number of initial studies have shown that AGPs are involved in responses to phytohormones such as abscisic acid (ABA), methyl jasmonate (MeJA), and gibberellin (GA). For example, AGP30 specifically responds to ABA concentration and subsequently influences seed germination in *Arabidopsis thaliana* [15]. AGP31 is repressed in *A. thaliana* plants treated with MeJA [16], and some AGPs affect the expression of gibberellin-induced genes in barley [17].

The importance of AGP involvement in the reproductive processes of flowering plants is reflected in studies of glycosylphosphatidylinositol-anchored proteins (GPI-APs) in the interaction between pollen/pollen tube and pistil tissues during pollination, such as pollen germination on the stigma, PT growth in the transmitting tract, PT guidance toward the ovule, and PT reception in the embryo sac [18]. Furthermore, a recent study has shown that AGPs are required for clathrin-mediated endocytosis of extracellular anchors [19]. Extracellular AGPs function as conserved receptors for clathrin-mediated endocytosis of anchors and traverse the plasma membrane to initiate endocytosis. Thus, AGPs may act as a nutrient source to participate in PT growth. However, the precise mechanism underlying their mode of action remains unclear.

Of great interest is the study of the role of AGPs in the pollen–pistil system and successful communication between the male and female gametophytes during sexual reproduction in plants. In this review, we systematically examine the structural diversity of AGPs and their functional significance, the role of AGPs at the stages of microsporogenesis and pollen grain wall formation, the mechanisms of AGP involvement in directed PT growth and signaling at different stages of pistil interaction, as well as the participation of AGPs in overcoming self-incompatibility barriers and reproductive barriers in distant hybridization.

## 2. Structural Organization of AGPs Underlying Reproductive Functions

AGPs are numerous highly glycosylated macromolecules belonging to the superfamily of hydroxyproline-rich glycoproteins. They consist of a protein backbone enriched in hydroxyproline, proline, alanine, serine, and threonine (the PAST domain), with covalently linked type II arabinogalactan chains that account for more than 90% of the molecular mass [20,21,22].

### 2.1. Protein Backbone: Structural Scaffold and Determinant of Glycosylation Pattern

The protein backbone of most AGPs is characterized by the presence of a PAST domain, an N-terminal signal peptide for secretion, and a C-terminal hydrophobic region to which a glycosylphosphatidylinositol anchor (GPI anchor) may be attached [7]. Following post-translational hydroxylation of proline residues, hydroxyprolines are formed, to which arabinogalactan chains are attached via O-glycosidic linkages. The composition of the protein backbone, enriched in Ala, Ser, and Thr residues, determines the density of O-glycosylation sites and, consequently, the level and pattern of protein modification. Depending on the structure of the polypeptide chain and the presence of additional domains, classical and chimeric AGPs are distinguished (Figure 1). The structure of the AGP backbone dictates the pattern and density of glycosylation, thereby defining the spatial organization of the molecule. This predetermines the subsequent localization of the protein in the cell wall and its involvement in reproductive processes, particularly in pollen–pistil interactions.

### 2.2. Arabinogalactan Glycans: Hydrated and Ca^2+^-Regulated Extracellular Matrix

The carbohydrate moiety of AGPs constitutes up to 90% of their molecular mass and is represented by type II arabinogalactan, comprising a (1→3)-β-D-galactose backbone with (1→6)-β-D-galactose side chains, often modified with arabinose. Rhamnose, mannose, xylose, glucose, fructose, and glucuronic acid may also be present, providing high structural variability of AGPs (Figure 1) [6,20,22]. The hydrophilicity and flexibility of the carbohydrate chains ensure the retention of water and ions, including calcium ions (Ca^2+^). The glycan structure influences cell recognition, adhesion, and signaling [23]. In reproductive processes, these properties are important for pollen hydration, PT growth, and regulation of cell wall plasticity through the management of Ca^2+^ distribution and their interaction with pectin components [12].

### 2.3. GPI Anchor: Spatial Organization and Signal Transduction

The GPI anchor is a component of classical AGPs, AG peptides, and FLAs [24] (Figure 1). The GPI anchor plays a role in signal transduction via protein release from its anchor by phospholipase action, which can generate intra- and extracellular messengers [25]. The GPI anchor underpins the ability of AGPs to participate in intercellular communication, ensures mobility within the membrane and the potential for involvement in signaling, and also allows for regulation of AGP secretion into the apoplast [18,20]. In the reproductive process, this is particularly important at the pollen–pistil interface, where rapid remodeling of signaling and adhesive interactions is required to ensure successful PT growth. However, the extent to which the GPI anchor of a given protein influences its function remains an open question.

### 2.4. Functional Domains: Expanding the Molecular Capabilities of AGPs

Chimeric AGPs contain additional functional domains, including fasciclin-, nodulin-, and xylogen-like domains, which expand their biological functions (Figure 1) [22,26]. The fasciclin domain confers upon AGPs the properties of cell adhesion molecules [27]. Through this domain, the proteins are capable of participating in cell-to-cell interactions and interactions with cell wall components, maintaining tissue mechanical stability, and regulating cell growth processes. In *Arabidopsis*, proteins with FAS domains can function as adhesion molecules [28]. FAS domains mediate intercellular interactions, adhesion, and signal transduction. The nodulin domain expands AGP involvement in signaling processes and intercellular communication [9]. Typically, proteins with this domain are associated with substance transport, ion exchange regulation, cell differentiation, and coordination of physiological processes. The xylogen domain enables AGP participation in cell wall remodeling and extracellular matrix organization [20]. It is assumed that this domain contributes to the regulation of tissue growth and spatial cell organization.

Thus, the functional properties of AGPs are determined not by a single structural element but by their modular organization. The protein backbone shapes the molecular architecture and determines the arrangement of glycosylation sites; arabinogalactan chains provide specific physicochemical properties and interactions with cell wall components and calcium ions; the GPI anchor controls membrane localization and involvement in signaling processes; and additional functional domains expand the spectrum of biological functions of individual family members. It is likely that various combinations of these structural components underlie the functional specialization of AGPs, allowing different proteins to participate in successive stages of pollen–pistil interaction. Immunolocalization using monoclonal antibodies (JIM8, JIM13, LM2) and the Yariv reagent confirms the widespread distribution of AGPs in reproductive tissues; however, due to cross-reactivity with common carbohydrate epitopes, these methods do not distinguish between individual genetic products [29].

Most studies focus on the presence or absence of AGPs, whereas the question of how exactly changes in carbohydrate chain structure (composition, length, degree of branching, modifications such as 4-O-methylation of glucuronic acid) affect function remains largely unexplored. Critically important is the identification of glycosyltransferases (GTs) and glycosidases responsible for the biosynthesis and modification of AGP glycans in reproductive tissues. Promising candidates include glucuronosyltransferases (GLCATs), which add GlcAc residues required for Ca^2+^ binding, as well as arabinosyltransferases and galactosyltransferases.

### 2.5. Regulation of AGP Expression: Upstream Regulators and Internal/External Cues

AGPs are encoded by large multigene families, and their expression is regulated primarily at the transcriptional level in a tissue-, cell-type-, and developmental-stage-specific manner. The best experimentally supported example in reproductive tissues is the transcription factor TEK (AT2G42940), which positively regulates the expression of *AGP6*, *AGP11*, *AGP23*, and *AGP40* in *Arabidopsis thaliana*. Mutations in *TEK* lead to impaired exine/nexine formation, demonstrating that AGPs act downstream of a defined transcriptional program during pollen wall development [30]. Beyond TEK, the upstream transcription factor network controlling AGP genes in the pollen, stigma, transmitting tract, ovule, and synergids remains largely unresolved. This is now explicitly highlighted as a major knowledge gap and a priority for future research.

Additional transcription factors are likely involved, but their identity remains largely unknown. post-translational modification machinery, including proline hydroxylation, O-glycosylation, and GPI-anchor addition; glycosyltransferases and glycosidases, such as GLCATs, galactosyltransferases, and arabinosyltransferases, which determine AGP glycan structure and Ca^2+^-binding capacity [31]; GPI-anchor biosynthesis genes, e.g., SETH1/SETH2, which affect pollen germination and tube growth [24]; local ionic and mechanical cues, including Ca^2+^, pH, ROS, and cell wall integrity, which modulate AGP function in the apoplast.

AGP genes are regulated by developmental, tissue-specific, and hormonal signals. internal factors include developmental stage, cell type, hormone signaling. Internal factors influencing AGP expression and function. phytohormonal signals, including ABA (e.g., AGP30 responds to ABA and affects seed germination [15]), methyl jasmonate (e.g., AGP31 is repressed by MeJA [16]), and gibberellin (some AGPs affect GA-induced gene expression in barley [17]); AGP expression and function can be modulated by environmental and physiological conditions, including pollination, humidity, temperature, abiotic stress, and hormone-related signals. For example, humidity affects premature pollen germination in mutants of intine-associated AGPs/FLAs, and pollination induces pistil-dependent changes in AGP gene expression in the male gametophyte. External factors include pollination, humidity, temperature, abiotic stress, and other environmental cues.

## 3. Role of AGPs in Successive Stages of Pollen–Pistil Interaction

### 3.1. Role of AGPs in the Formation of a Functional Male Gametophyte

The process of male gametophyte formation proceeds within the anthers in close interaction with the surrounding tapetum. AGPs participate in this process at all key stages of microsporogenesis and microgametogenesis [9] (Table 1). Immunolocalization using monoclonal antibodies (JIM8, JIM13, MAC204) has shown that AGPs are already present in the cell walls of pollen mother cells, then in tetrads, in the exine cavities of free microspores, and in the walls of the generative cell and sperm cells [32]. Thus, AGPs accompany male gametophyte development from meiosis to the mature PG, indicating their fundamental role in structural differentiation and intercellular signaling at all stages.

The mature pollen grain consists of a vegetative cell, which develops into the PT, and two sperm cells, which will fuse with the egg cell and the central cell, giving rise to the embryo and endosperm, respectively [61]. The PG has a complex wall consisting of the outer exine (comprising sexine and nexine) and the inner intine [9,31,62] (Figure 2).

The exine provides protection and interaction with the stigma, while the intine is involved in germination and PT growth [63]. Disruptions in the formation of either of these layers lead to male sterility [64]. Numerous genetic studies have identified a range of pollen-specific AGPs, mutations in which cause similar defects—abnormalities in the exine and/or intine (Table 1).

Lysine-rich AGPs constitute a distinct subgroup of classical AGPs characterized by a short lysine-rich region within the AGP domain. In Arabidopsis thaliana, this subgroup comprises AtAGP17, AtAGP18 and AtAGP19, all of which possess a C-terminal GPI-anchor addition sequence. These proteins exhibit distinct tissue-specific expression patterns, with AtAGP18 being particularly abundant in flowers. Functional studies have demonstrated that lysine-rich AGPs are involved in plant reproductive development. For example, suppression of AtAGP18 affects female gametophyte development, whereas loss of AtAGP19 results in defects in plant growth and reduced seed production. These findings indicate that the lysine-rich AGP subgroup may contribute to reproductive development through regulation of cell growth, differentiation and cell–cell interactions [25,40]. Compared with classical AGPs and FLAs, Lys-rich AGPs remain poorly studied in plant reproduction; this as an important direction for future research.

#### 3.1.1. Critical Stages of AGP Action: From Exine Formation to Intine Construction

In *Arabidopsis thaliana*, several AGPs required for pollen development have been identified: classical AGP6, AGP11, AGP24, fasciclin-like FLA3, FLA14, as well as AG peptides AGP23 and AGP40 [65,66] (Figure 2, Table 1). Notably, regardless of the subfamily, most mutants in these genes exhibit disruption of PG wall integrity, manifested as thinning or absence of the nexine, improper sporopollenin deposition, or intine defects. This suggests that AGPs perform two interconnected functions at different temporal stages: an early function (exine formation) and a late function (intine formation).

#### 3.1.2. Early Role: Exine Formation and Communication with the Tapetum

At early stages (tetrads, free microspores), AGPs act as signaling molecules regulating the interaction between microspores and the tapetum, which supplies sporopollenin precursors for the exine. Direct evidence: the transcription factor TEK (AT2G42940) regulates the expression of *AGP6*, *AGP11*, *AGP23*, *AGP40*, and *TEK* mutations lead to impaired nexine formation [67] (Figure 2, Table 1). This indicates that AGPs are downstream effectors of the genetic program for exine construction. In rice, the chimeric AGP MTR1 (an ortholog of AtFLA20) is critical for timely tapetum vacuolization and microspore formation—its mutants exhibit abnormal microspore morphology and male sterility [48]. Similarly, in rice, OsFLA1 positively regulates exine development, and its knockout leads to shriveled pollen [49]. Thus, AGPs at early stages ensure the structural integrity of the exine through tapetal support and sporopollenin deposition.

#### 3.1.3. Late Role: Intine Formation and Preparation for Germination

In the late stages (mature pollen), AGPs participate in the construction of the intine—the inner layer rich in pectins and cellulose, which is critical for hydration and PT emergence. In *Arabidopsis*, FLA3 and FLA14 affect intine formation (Figure 2, Table 1): *FLA3* mutants have approximately 50% abnormal pollen with inner layer defects [45], while overexpression of FLA14 produces shriveled PGs and reduces fertility [47]. In Chinese cabbage, BrFLA2, BrFLA28, and BrFLA32 are required for proper germination timing and, likely, for intine formation, since their suppression causes premature germination under high humidity conditions [68]. In *Brassica campestris*, the genes *BcMF8* and *BcMF18* (orthologs of AGPs) are also involved in intine formation: mutations in *BcMF8* lead to abnormal intine development and ectopic apertures, while *BcMF18* is required for normal cellulose distribution in the intine [69]. This underscores the conserved role of AGPs in intine biogenesis across different species.

#### 3.1.4. AGPs as Mediators of Intercellular Signaling Between the Microspore and the Tapetum

Through their GPI anchors, AGPs are localized on the surface of microspores and tapetal cells. Their carbohydrate chains are capable of binding signaling molecules (e.g., peptides or small proteins) and initiating cascades that regulate the expression of genes responsible for the synthesis of exine components [18]. Disruption of this signaling (as in mtr1 or tek mutants) leads to sporopollenin defects and improper exine layer formation [48,67].

#### 3.1.5. AGPs as Structural Organizers of the Matrix for Polysaccharide Deposition

In the intine, AGPs interact with pectins and cellulose via calcium bridges, creating a hydrated three-dimensional network necessary for the deposition of new layers [12]. Their ability to bind Ca^2+^ [70] is critical for controlling local ion concentration, which affects synthase activity and the sol–gel transition state in the matrix. Mutants lacking specific AGPs (*FLA3*, *FLA14*, *BcMF18*) lose the ability to form a normal intine, manifesting as thinning, loss of elasticity, or premature germination [64].

#### 3.1.6. AGPs as Regulators of Polarity and Germination Timing

At late stages, AGPs (e.g., FLA14, BrFLAs) are involved in maintaining pollen quiescence until deposition onto the stigma. Their absence leads to premature activation, indicating that AGPs transmit signals regarding humidity and mechanical wall status, thereby controlling germination readiness [47,61]. Thus, despite the diversity of AGPs involved in pollen development (from classical to chimeric FLAs), their common function boils down to ensuring proper PG wall architecture through signaling and structural mechanisms. All described AGP mutants exhibit a similar outcome—reduced fertility due to exine or intine defects—confirming the indispensability of these glycoproteins at all stages of male gametophyte ontogeny [45,47].

An open question remains regarding specificity: why do different AGPs produce distinct phenotypes (exine vs. intine) despite apparent functional similarity? This may be explained by their differential spatiotemporal expression profiles, cell-type-specific localization, quantitative expression levels, and structural heterogeneity of their carbohydrate chains, which collectively determine interactions with specific partners (receptors, enzymes, or polysaccharides).

#### 3.1.7. Model: AGPs as an Integral Scaffold for Cellulose and Callose Deposition

GPI-anchored AGPs on the plasma membrane surface create specialized microdomains that serve as both a physical matrix and a signaling hub. By binding Ca^2+^ and interacting with pectins, AGPs establish a local environment favorable for the activity of cellulose and callose synthases [31,48]. In this presumed model, AGPs may act not as passive structural elements but as active organizers, determining the initiation sites, direction, and rate of deposition of new cell wall layers critically important for intine and exine formation.

#### 3.1.8. Orthologs and Conservation of Functions Across Other Species

Comparative studies indicate that AGP functions in pollen development are conserved. In rice, *OsAGP7* and *OsAGP10*—orthologs of *AtAGP6/AtAGP11*—are expressed in pollen, though their precise mechanisms are still under investigation [71]. In Brassica, homologs BcMF8 and BcMF18 have been identified [69] (Table 1), as well as several AGPs differentially expressed in fertile and sterile flower buds [72]. This indicates that the key role of AGPs in pollen wall formation is evolutionarily ancient and conserved among angiosperms, although individual genes may acquire specialized functions depending on the species.

## 4. Role of AGPs in the Process of Germination and Growth of the Male Gametophyte in Pistil Tissues

### 4.1. Adhesion and Hydration of the Pollen Grain on the Stigma

Due to their high degree of glycosylation, AGPs are capable of performing a wide range of functions in the reproductive structures of angiosperms. In the PT and pistil tissues (stigma, style, ovary), they participate in both structural organization and signaling interactions, ensuring successful communication between the male and female gametophytes.

Upon deposition of the PG onto the stigma surface, contact is established between its surface and the papilla cells. This interaction leads to the formation of an adhesive structure—the so-called pollen “foot” (Figure 3). An adhesive matrix containing pectins and AGPs from both pollen and stigma is formed at the contact zone. AGPs bind to pectin polysaccharides via calcium cross-links, forming molecular bridges [11,23,31], thereby ensuring stable intercellular interaction and species-specific recognition.

Specific AGPs in the stigmatic exudate create a hydrating and nutritive microenvironment, ensuring water and Ca^2+^ ion availability for pollen activation. Cell wall pectins, including homogalacturonans, form a flexible medium for adhesion and hydration [73]. Secretion of AGPs by papilla cells strictly correlates with pistil maturity and its readiness for pollination, as demonstrated in apple [74], magnolia [75], *Trithuria* [76], oak [77] and tomato [78]. Among specific markers, AGP1 is abundantly expressed in the stigma and participates in initial adhesion, whereas AGP12 is specific to papillae and serves as a marker of the mature pistil [66].

PG germination begins with hydration of the wall at the aperture region [79], where the wall is thinner and more amenable to expansion. At this site, AGPs form a ring between the aperture and the future PT tip [34] (Figure 3), preparing the zone for polar growth. It is now well established that AGP6 and AGP11 are functionally required for pollen germination [76], but it is not known which specific AGPs localize around the aperture prior to PT emergence. During pollination, both the PG and stigma cells exhibit an increase in cytosolic Ca^2+^ with different temporal dynamics. Lamport et al. (2014) [70] proposed that AGPs may act as periplasmic Ca^2+^ condensers, releasing Ca^2+^ in response to a proton flux and integrating it into the initial oscillations required for germination activation. This hypothesis is supported by recent data on the functional necessity of Ca^2+^ binding via glucuronic acid (GlcAc) residues for male fertility [31]. Thus, at the adhesion and hydration stage, AGPs perform a dual role—structural (formation of the adhesive matrix) and signaling (provision of the calcium signal for germination initiation).

### 4.2. Pollen Tube Growth in the Pistil Transmitting Tissues

#### 4.2.1. Molecular Mechanisms of PT Growth and the Role of AGPs

After germination, the PT penetrates the pistil tissues and begins its journey through the transmitting tract—a specialized secretory tissue rich in extracellular matrix, as found in Solanaceae (Figure 4), or within the hollow canal of the pistil, as in Liliaceae [80]. PT growth within the pistil is constrained by the extracellular matrix and the transmitting tissue [3,9,81,82]. Direct contact between the growing PT tip and the cell walls of the matrix is required for proper guidance.

PT tip growth is a polarized process dependent on the coordinated action of the actin cytoskeleton, exocytosis, and endocytosis [83]. A key role is played by the cytosolic Ca^2+^ gradient focused at the tip. AGPs participate in the formation of this gradient, acting as periplasmic Ca^2+^ condensers. According to the Hechtian oscillator model [84], AGPs bind Ca^2+^ via pairs of glucuronic acid residues; upon transmission of tension from the wall through Hechtian adhesion sites, H^+^-ATPase and mechanosensitive Ca^2+^ channels are activated, leading to local Ca^2+^ release and exocytosis activation. This model explains the oscillatory nature of PT growth and links the mechanical properties of the wall with ion fluxes, with AGPs acting as active regulators governed by pH and tension [84].

Transcriptomic studies, first performed on *Arabidopsis* pollen [85,86], demonstrated that pollen expresses a unique subclass of AGP genes, with some being induced only during pollen tube growth through pistil tissues. These data indicate pistil-dependent regulation of AGP expression and confirm that interaction with female tissues is a central event modulating the transcriptional landscape of the male gametophyte. Later transcriptomic analyses of *agp6*, *agp11* mutants revealed extensive genetic interactions, involving significant changes in the expression of genes related to cell wall biosynthesis and signaling pathways, indicating a broader regulatory network in which AGPs are involved [87,88].

In contrast to transcriptomes, which only indicate potential capabilities, proteomic data directly reveal the proteins actually present in the wall at a given moment. Studies have shown that AGPs constitute a significant fraction of PT cell wall proteins, with their composition dynamically changing depending on the growth stage [89,90].

#### 4.2.2. AGPs of the Pistil Transmitting Tissue as Regulators of Pollen Tube Growth

The extracellular matrix of the transmitting tract, rich in pistil-specific AGPs, supports and provides nutrition and adhesion for PT growth and guidance. The TTS protein is required for normal PT growth. TTS acts as a nutrient source and as an adhesion molecule for PT growth. This protein promotes PT growth and acts as a chemical attractant for the PT [80]. TTS is secreted into the transmitting tissue and forms a glycosylation gradient along the style (Figure 4B). Notably, the degree of glycosylation increases toward the ovary [91,92]. The PT appears to absorb TTS: the protein binds to the PT surface, then becomes localized in its cell wall and is gradually deglycosylated [53]. In this way, TTS proteins form the extracellular environment along which the PT grows. Although the role of TTS in supporting PT growth is well established, the molecular mechanisms of its perception remain largely unexplored. In particular, no TTS receptors have been identified to date; it is not known whether TTS interacts directly with PT AGPs or other cell wall components, nor how its deglycosylation and signal transduction are achieved. These questions represent one of the most promising directions for future research on AGPs in plant reproductive biology.

In addition to TTS, the pistil extracellular matrix contains other pistil-specific proteins that regulate PT growth, promoting either fertilization or rejection: pistil-specific extensin-like protein III (PELPIII) and a 120-kDa glycoprotein (120 K) [58] (Figure 4E,F).

#### 4.2.3. Genetic and Immunological Data on AGPs in the PT

In *Arabidopsis thaliana*, the genes *AGP6* and *AGP11* are critical for pollen germination and PT development [34,66,88] (Table 1). Their ortholog in rice (OSIAGP) is also active at these stages. In pear, knockdown of *PbrAGP1* and *PbrAGP5* inhibits PT growth [36]; in oil camellia, CoFLA7 enhances PT elongation [46]. In *B. rapa*, BrAGP11.1 is required for germination, while BrAGP15.3 and BrAGP54.1 exhibit expression patterns similar to pollen AGP [35]. Transcriptomic data show that pollen expresses a unique set of AGP genes, with some being induced only during growth through the pistil [85,86].

Immunolocalization using monoclonal antibodies (JIM13, MAC207, JIM8, JIM4) has revealed dynamic patterns of AGP distribution. JIM13 labels AGPs at the apex of the growing PT, with the signal intensifying upon growth activation; MAC207 intensely stains the tube emergence zone; JIM8 binds to AGPs of mature pollen but disappears upon germination [34,93]. Various patterns have been described in the PT cell wall: in *N. tabacum*, JIM13 gives uniform staining, whereas MAC207 does not [94]. Pectinase treatment reveals periodic ring-like AGP deposits along the tube [94]. Differences in labeling patterns may be due to species specificity, epitope accessibility, and pistil type (dry/wet, solid/hollow style), indicating the complexity of AGP organization in the cell wall [95].

Experiments with the Yariv reagent—a specific AGP inhibitor—have confirmed their functional significance: treatment of *L. longiflorum* PTs with active Yariv reagent arrested growth without halting vesicular secretion, but disrupted wall architecture; removal of the reagent restored growth [96]. In *N. tabacum*, high concentrations of Yariv reagent also reduced growth [93]. These data indicate that AGPs are required for proper deposition of new cell wall layers during PT elongation.

### 4.3. Navigation to the Embryo Sac and Pollen Tube Reception

#### 4.3.1. Molecular Navigation Signals

After passing through the transmitting tract, the PT must precisely navigate toward the micropyle of the ovule, enter the synergid, and rupture to release the sperm cells—a process termed PT reception. At this stage, AGPs participate in both attraction and control of polyspermy (Figure 4D). In *Torenia fournieri*, a 4-O-methyl glucuronosyl arabinogalactan (AMOR) has been identified, which induces competence in the PT to respond to attractant LURE peptides secreted by synergids [97,98]. AMOR is a carbohydrate moiety related to AGP glycans and serves as an important signal that primes the PT for final guidance. High concentrations of Ca^2+^ and AGPs have been detected in the synergid cells and filiform apparatus of the embryo sac [99,100], consistent with the role of AGPs as sources of Ca^2+^ for directed growth.

#### 4.3.2. Role of AGPs in Reception and Prevention of Polyspermy

A group of early nodulin-like proteins (ENODLs), containing an arabinogalactan glycomodule, are specifically expressed in the micropylar region of synergids in *Arabidopsis*. These proteins (EN11–EN15) are localized on the plasma membrane of synergids, particularly at the filiform apparatus, and are required for PT reception (Figure 4D) [50,51,101]. Their precise molecular mechanism has not yet been established, but they are thought to participate in recognition of the PT tip and initiation of tube rupture.

The classical AGP4 (JAGGER) in *A. thaliana* prevents the growth of multiple PTs into a single embryo sac (Figure 4D). In *jagger* mutants, an increased number of polytubes and persistent synergids that fail to degenerate are observed, leading to polyspermy. AGP4 is localized at the periphery of the ovule integuments and in cells of the transmitting tract [37,102]. Thus, AGP4 acts as a negative regulator of attraction, ensuring monogamous fertilization of each ovule.

Other AGPs expressed in ovules also affect fertility (Figure 4D): AGP18 is involved in the selection of viable megaspores [39], while AGP19, expressed in the style, ovary wall, and transmitting tract, influences silique and seed production [40]. Despite these data, the molecular mechanisms linking AGPs to receptor kinases or ion channels at the final stages remain the least understood. In particular, no direct receptor for AGPs on the surface of the PT or synergids has been identified, making this a priority direction for future research.

Thus, it can be concluded that AGPs perform three sequential groups of functions: on the stigma—adhesion, hydration, and germination initiation; in the transmitting tract—maintenance of apical growth, wall plasticity, and interaction with pistil-specific signaling molecules; in the embryo sac—navigation, reception, and control of polyspermy. This division reflects both the chronology and the changing molecular roles of AGPs in the reproductive success of angiosperms.

## 5. Role of AGPs in Self-Incompatibility Mechanisms

Self-incompatibility (SI) is a critical prezygotic barrier that prevents self-pollination and promotes the maintenance of genetic diversity within populations [103]. Interspecific incompatibility (InInc), in turn, restricts gene flow between species, preserving their genetic integrity [5]. In angiosperms, there are two main genetic systems of SI: sporophytic (characteristic of Brassicaceae), where the pollen phenotype is determined by the genotype of the maternal sporophyte, and gametophytic (found in Solanaceae, Rosaceae, and Plantaginaceae), where the pollen phenotype is determined by its own haploid genotype [104].

AGPs are widely represented in reproductive tissues, and their abundance in stigmatic exudates, the transmitting tract, pollen grains, and tubes indicates their potential role in recognition and gametophyte selection processes. Current evidence allows AGPs to be considered important modulators of SI; however, the nature of their involvement differs fundamentally between the two main types of this system.

### 5.1. Sporophytic Self-Incompatibility (Brassicaceae)

Brassicaceae, SI is controlled by the polymorphic S locus, which contains three tightly linked genes: the S-locus receptor kinase (SRK)—the female determinant, the S-locus cysteine-rich protein (SCR/SP11)—the male determinant, and the S-locus glycoprotein (SLG). Interaction of SCR/SP11 with the extracellular domain of SRK on stigma papilla cells triggers a signaling cascade leading to rejection of incompatible pollen (Figure 4G) [103].

The role of AGPs in sporophytic SI remains the least studied compared to the gametophytic system; however, there are indications of their possible involvement. As early as the 1970s, it was shown that mature stigmas of *Brassica oleracea* contain large amounts of a glycoprotein absent at earlier developmental stages, which may play a role in pollen–stigma interactions. This glycoprotein appears to be an AGP-like molecule, as its appearance correlates with the acquisition of SI competence in the stigma [105]. However, identification of specific AGPs directly involved in SI signaling in Brassicaceae has not yet been completed.

Based on available data, a hypothetical model can be proposed in which AGPs on the surface of stigma papilla cells may: form an extracellular matrix that modulates the accessibility of SCR/SP11 to SRK; act as co-receptors or adaptor molecules facilitating ligand–receptor interaction; and participate in the regulation of calcium signals and reactive oxygen species (ROS), which mediate early responses to incompatible pollen. Supporting the latter hypothesis is the ability of AGPs to bind Ca^2+^ and act as calcium condensers [70,84], as well as evidence that during the SI response in Brassicaceae, a rapid Ca^2+^ influx into papilla cells is observed [106]. Thus, AGPs may be part of an apoplastic signaling network modulating the ionic changes required for pollen rejection. Nevertheless, direct genetic or biochemical evidence for the involvement of specific AGPs in sporophytic SI is currently insufficient, and this represents a promising direction for future research.

### 5.2. Gametophytic Self-Incompatibility (Solanaceae)

In contrast to the sporophytic system, gametophytic SI in Solanaceae is based on S-RNases—style ribonucleases encoded by the S locus—which degrade the rRNA of incompatible pollen tubes, arresting their growth. A key feature of this system is that S-RNase itself does not possess species-specific rejection specificity—it requires additional factors, and it is precisely here that AGPs perform the function of SI modulators.

#### 5.2.1. Pistil-Specific AGPs as Co-Factors of S-RNase

*N. tabacum* and *N. alata*, three pistil-specific glycoproteins possessing AGP-like properties have been identified: TTS (Transmitting Tissue Specific), PELPIII (class III pistil-specific extensin-like protein), and the 120-kDa glycoprotein [80]. All three proteins are localized in the extracellular matrix of the style transmitting tissue and are capable of binding S-RNases in vitro. This binding suggests that AGPs form functional complexes with S-RNase in the extracellular matrix, which then interact with growing pollen tubes.

#### 5.2.2. Differential Roles of AGPs in Self- and Interspecific Incompatibility

Despite their structural similarity, these three proteins perform different functions. TTS is involved in stimulating PT growth, acting as a chemoattractant and nutrient source [3,81]. Its role in SI is indirect—it provides optimal conditions for the growth of compatible tubes but is not a direct effector of rejection. The 120-kDa glycoprotein is required for S-specific rejection of incompatible pollen. Knockdown of 120-kDa protein expression leads to impaired S-specific rejection, indicating its critical role as a co-factor of S-RNase. Interestingly, the 120-kDa protein exhibits significant size polymorphism (from 80 to 120 kDa) between different compatible and incompatible plants, consistent with its involvement in allele-specific recognition. PELPIII is required for InInc, rather than for SI. In *N. tabacum* plants with suppressed PELPIII expression, loss of PT growth inhibition of *N. obtusifolia* and *N. repanda* was observed, whereas rejection of self-pollen remained intact [55,107]. Thus, PELPIII appears to provide a species-specific barrier preventing interspecific fertilization.

Phylogenetic analysis has shown that the genes *PELPIII*, *120 K*, *TTS*, and *NtPRP* originated from a common ancestral AGP and diversified through duplications [58]. It is hypothesized that the ancestral gene was involved in supporting PT growth through the style. Subsequent duplications gave rise to a gene responsible for SI (the precursor of 120 K) and a gene responsible for interspecific incompatibility (the precursor of PELPIII). Such evolutionary diversification reflects the adaptation of AGPs to perform specialized functions in reproductive isolation systems.

Thus, the role of AGPs in self-incompatibility is system-dependent. In sporophytic SI (Brassicaceae), the involvement of AGPs remains hypothetical and requires further experimental confirmation. In gametophytic SI (Solanaceae), AGPs act as obligatory co-factors of S-RNase, ensuring S-specific rejection (120 K) (Figure 4E) and InInc (PELPIII) (Figure 4F). This distinction underscores the remarkable plasticity of AGPs as signaling molecules, capable of adapting to different molecular mechanisms of SI across diverse angiosperm taxa.

Gene duplications, as shown for pistil-specific AGPs in Nicotiana (giving rise to *TTS*, *PELPIII*, *120-kDa* protein, and *NtPRP*), represent a powerful evolutionary mechanism for the emergence of new functions [58]. However, it remains unclear exactly how these duplications led to the complexity of signaling pathways and the appearance of specialized roles in SI and InInc. Comparative genomic and transcriptomic (phylogenomic) studies of AGPs across different angiosperm taxa, particularly among species with different SI systems, may help identify patterns of co-evolution of AGPs with other SI components (e.g., S-RNases in Solanaceae vs. Brassicaceae). Also of interest is the question of whether AGP-specific signaling cascades that are independent of classical SI pathways exist.

#### 5.2.3. Role of AGPs in the Functioning of Reproductive Barriers in Distant Hybridization

Direct experimental evidence indicating the involvement of arabinogalactan proteins in the functioning of reproductive barriers during distant hybridization remains limited. Nevertheless, the results of studies on the role of AGPs in plant reproductive processes suggest their potential participation in mechanisms of interspecific reproductive incompatibility. AGPs are localized on the surface of pollen grains and pollen tubes, as well as in pistil tissues, and are involved in intercellular signaling, cell wall organization, and interactions between male and female reproductive tissues [102,108]. The importance of regulating cell wall properties for overcoming interspecific reproductive barriers is supported by studies on the molecular mechanisms of distant hybridization in the genus Magnolia, in which the PME4 gene, encoding a pectin methylesterase, was linked to increased efficiency of interspecific crosses [109]. Through their functions in cell wall organization and intercellular interactions, AGPs are potentially capable of influencing pollen recognition and adhesion, germination, growth, and directed progression of the pollen tube [38,52]. Since disruptions of these processes can lead to reduced interspecific compatibility, AGPs may be considered potential molecular participants in the formation of prezygotic reproductive barriers during distant hybridization. However, this assumption requires further experimental verification.

## 6. Conclusions and Future Perspectives

Arabinogalactan proteins (AGPs) represent a unique family of highly glycosylated macromolecules that play a fundamental role in the reproductive processes of angiosperms. Their complex structure, comprising a proline/hydroxyproline-rich protein backbone, covalently linked type II arabinogalactan chains, and, in most representatives, a GPI anchor, endows them with exceptional functional plasticity. It is the combination of three key properties—hydrophilicity and Ca^2+^-binding capacity, interaction with pectins via calcium bridges, and GPI-mediated dynamic localization—that transforms AGPs into universal mediators between the cell wall, the plasma membrane, and intracellular signaling cascades.

Throughout the entire reproductive cycle—from PG formation to sperm cell delivery to the embryo sac—AGPs perform sequential and interconnected functions. At the early stages of microsporogenesis, AGPs, including classical AGP6/AGP11, AG peptides AGP23/AGP40, and fasciclin-like FLA3/FLA14, participate in the formation of the exine and intine, ensuring the structural integrity of the pollen grain and its readiness for germination. At the stage of interaction with the pistil, AGPs fulfill a dual role—structural (pollen adhesion and hydration on the stigma, maintenance of tube wall plasticity in the transmitting tract) and signaling (involvement in compatibility recognition, directional navigation, and tube reception in the embryo sac).

Of particular note is the role of AGPs in self-incompatibility systems: in gametophytic SI of Solanaceae, AGPs (120-kDa protein and PELPIII) act as mandatory co-factors of S-RNase, ensuring S-specific rejection and InInc. The conservation of key AGP functions across different species—from *Arabidopsis* and *Brassica* to *Oryza* and *Nicotiana*—attests to the evolutionary antiquity of this protein group and their fundamental role in the reproductive biology of angiosperms. At the same time, interspecific diversification of AGP genes (e.g., duplications that gave rise to *TTS*, *PELPIII*, and the *120-kDa* protein in *Nicotiana*) demonstrates the remarkable adaptability of these molecules to various mechanisms of reproductive isolation.

The rapid development of omics technologies, as well as CRISPR/Cas9 technology, has revolutionized the functional study of AGPs. In contrast to traditional T-DNA insertion mutants, which often yield weak or lethal phenotypes due to functional redundancy of AGPs, CRISPR enables the generation of multiple knockouts in a single generation (e.g., higher-order mutants for several *GALT* genes), opening up the possibility of studying functional redundancy and uncovering hidden phenotypes [110]. Furthermore, CRISPR editing has allowed the introduction of point mutations into glycosylation sites or GPI-anchor domains, making it possible to dissect specific molecular functions of AGPs in vivo. The application of CRISPR to generate mutants in genes encoding glycosyltransferases responsible for AGP glycan biosynthesis will lead to the discovery of new mechanisms regulating PT growth. Integration of transcriptomic, proteomic, and genomic data with CRISPR mutant phenotypes enables the construction of multi-level models of AGP-mediated regulatory processes. Such a systems approach is particularly promising for understanding how glycosylation dynamics and interactions with partner proteins ensure adaptation of pollen tube growth to changing conditions within pistil tissues.

Understanding the role of AGPs in interspecific incompatibility opens fundamentally new opportunities for biotechnology. Given that PELPIII in *Nicotiana* is involved in the rejection of PTs from closely related species, it can be hypothesized that manipulation of AGP expression or glycosylation could “trick” the recognition system and overcome incompatibility barriers during interspecific hybridization. This is particularly relevant for fruit crops (e.g., apple, pear, citrus) and cereals (rice, wheat), where interspecific hybridization is limited by reproductive barriers.

Furthermore, a full understanding of AGP regulatory mechanisms will require in-depth research in the following areas: single-cell transcriptomics and spatial transcriptomics of reproductive tissues; ATAC-seq and ChIP-seq to identify cis-regulatory elements and upstream transcription factors; CRISPR/Cas9-based functional screens of candidate regulators; glycomics and glycoproteomics to link AGP glycan structure to function; and comparative analyses across species with different SI and interspecific incompatibility systems.

Thus, elucidating the structure of AGP–receptor complexes, deciphering the dynamics of glycosylation, and understanding the evolutionary pathways of AGP diversification not only deepen fundamental knowledge of plant reproductive biology but also open new horizons for biotechnological applications, including the overcoming of interspecific barriers and the creation of hybrid forms with improved agronomically valuable traits.

## Figures and Tables

**Figure 1:**
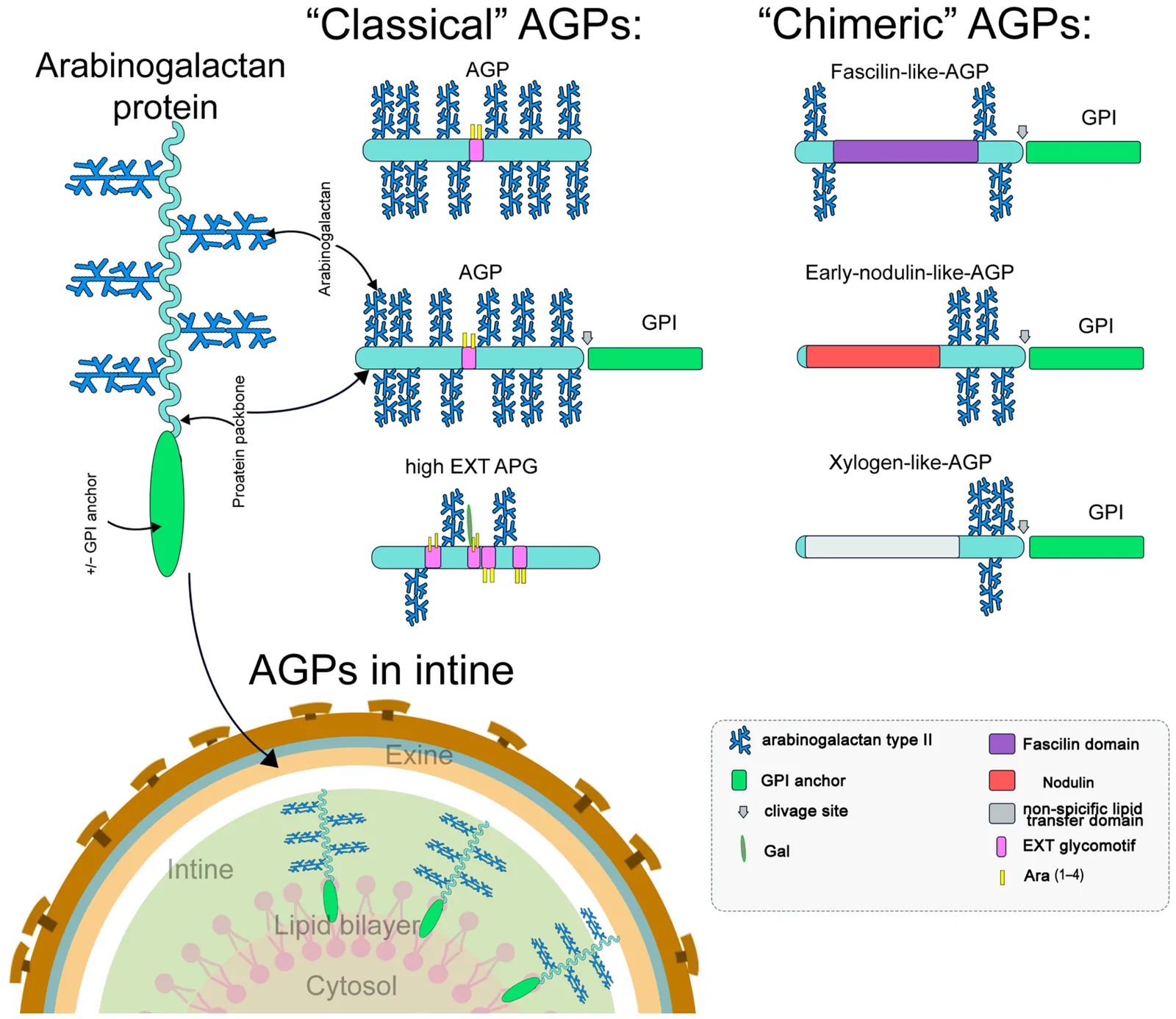
Schematic representation of AGP structure and their anchorage in the plasma membrane of the pollen grain via a GPI anchor. Classical AGPs and chimeric AGPs.

**Figure 2:**
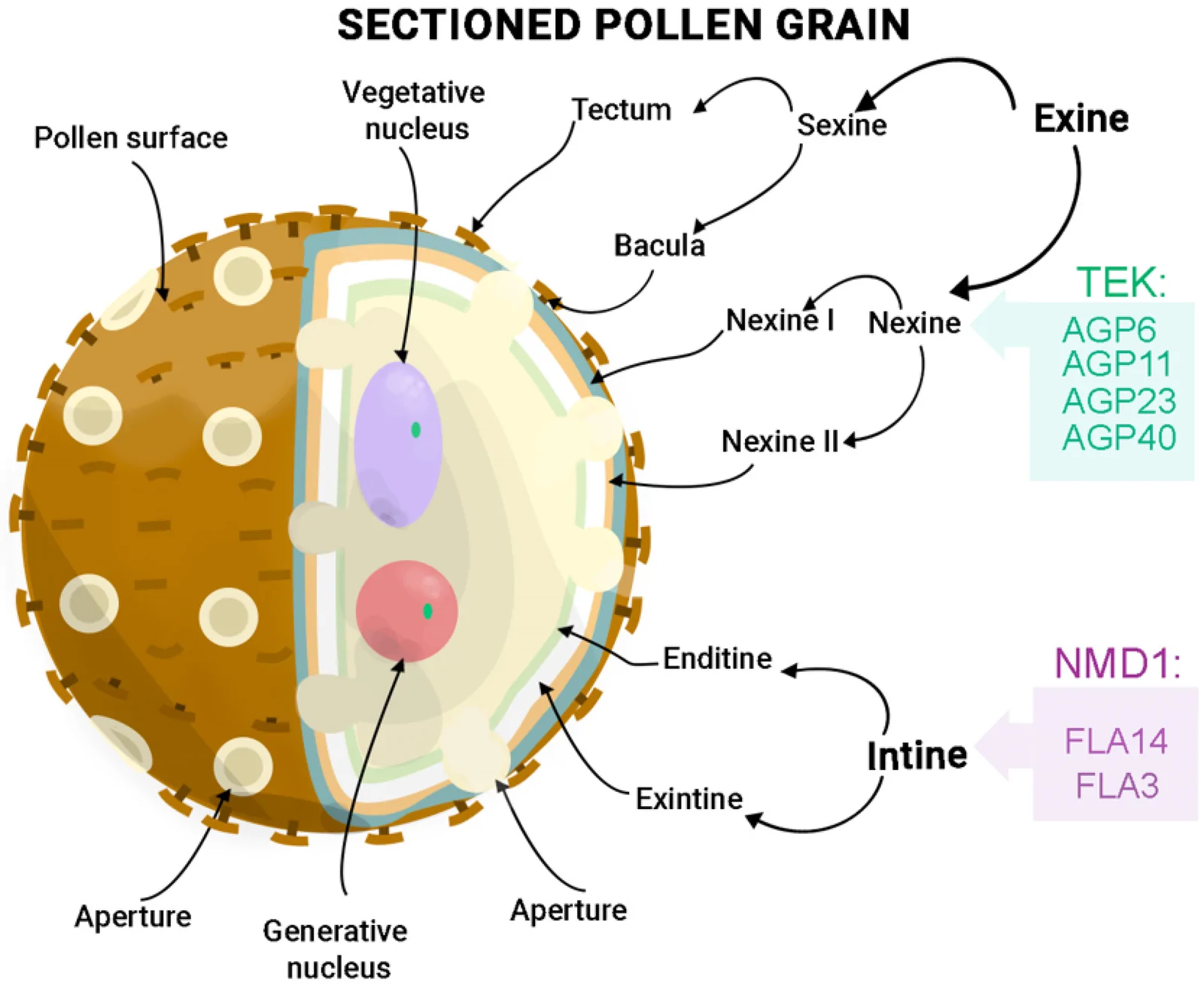
Schematic representation of the pollen grain indicating the known arabinogalactan proteins involved in the formation of its walls.

**Figure 3:**
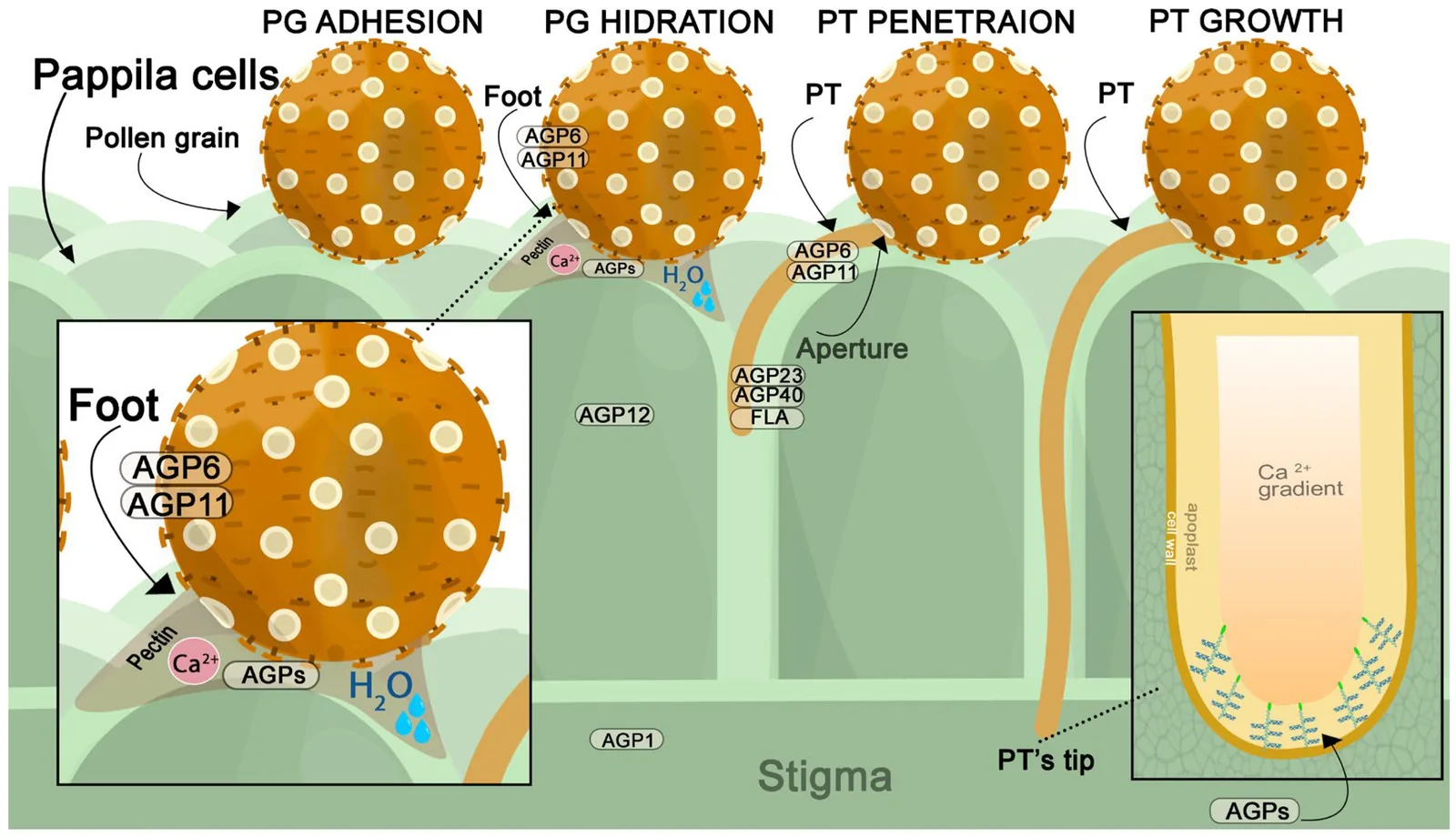
Schematic representation of the role of AGPs in the processes of adhesion, hydration, and germination of the pollen grain on the stigma of Brassicaceae.

**Figure 4:**
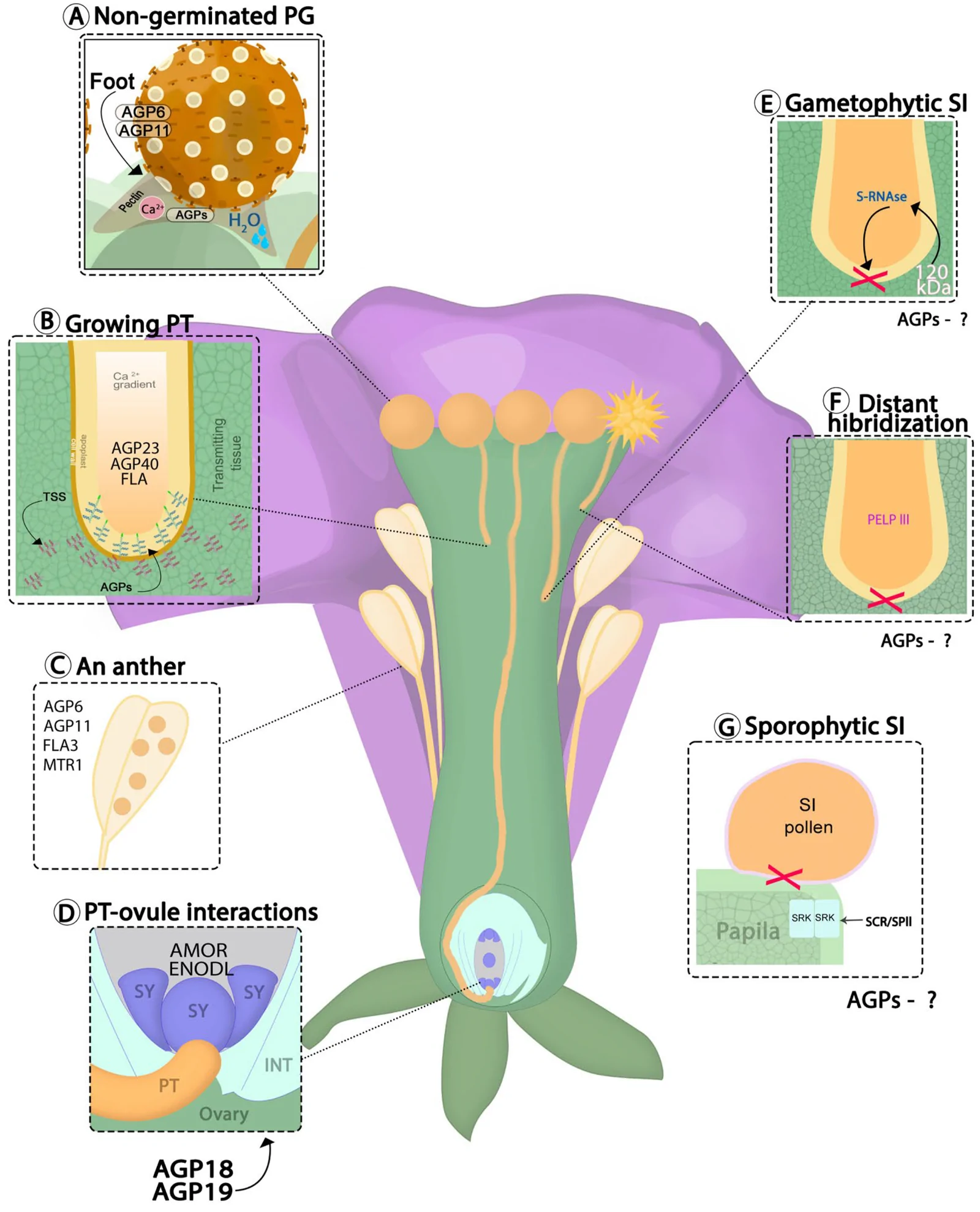
Schematic representation of the role of AGPs in male and female floral organs and in the process of pollen tube growth through pistil tissues. (**A**) Non-germinated PG—pollen-specific AGPs required for adhesion and hydration of the PG on the stigma; (**B**) Growing PT—pollen- and pistil-specific AGPs required for normal growth and navigation of the PT in the pistil transmitting tissue; (**C**) Anther—pollen-specific AGPs required for normal pollen formation and development; (**D**) PT–ovule interactions—pistil-specific AGPs required for successful fertilization; (**E**) Gametophytic SI—pistil-specific 120 K plays a direct role in S-specific pollen rejection. 120 K is internalized by the PT, forming complexes with S-RNases and other proteins. The role of other AGPs is unknown; (**F**) Distant hybridization—pistil-specific PELPIII specifically inhibits PT growth during InInc. It accumulates in the transmitting tissue of the extracellular matrix. After pollination, PELPIII moves from the cellular matrix into the walls of both compatible and incompatible pollen tubes. The role of other AGPs is unknown; (**G**) Sporophytic SI—Interaction of SCR/SP11 with SRK on stigma papilla cells, leading to rejection of incompatible pollen. The role of AGPs is unknown. Red cross indicates arrest of germination/PT growth.

**Table 1:** Classification of AGPs, structural features, and examples involved in reproductive processes.

Subfamily/Type	Structural Features of Protein	Gene Examples (Species)	Localization/Expression	Mutant Phenotype/Proposed Function	References
Classical AGPs	PAST-rich protein backbone, GPI anchor, O-glycosylation with Hyp-AG chains data	*AGP6*, *AGP11* (*A. thaliana*)	Pollen, developing PT	Defects in pollen germination and PT growth	[33,34]
		*BrAGP11.1*, *BrAGP15.3*, *BrAGP54.1* (*B. rapa*)	Pollen, PT, fertilization processes	Impaired germination, intine abnormalities	[35]
		*PbrAGP1*, *PbrAGP5* (pear)	Pollen, PT	PT growth inhibition upon knockdown	[36]
		*AGP4 (JAGGER)* (*A. thaliana*)	Ovule integuments, transmitting tract	Polyspermy (multiple PTs into one embryo sac)	[37,38]
		*AGP18* (*A. thaliana*)	Ovule (meiotic cells)	Involvement in viable megaspore selection	[39]
		*AGP19* (*A. thaliana*)	Style, ovary wall, transmitting tract	Reduced fertility, fewer siliques and seeds	[40]
AG peptides	Short (10–15 amino acids) PAST backbone, GPI anchor data	*BAN102* (*B. rapa*)	Pollen, PT	Homolog of AtAGP23, presumably involved in germination	[41]
		*OSIAGP* (*O. sativa indica*)	Germinating pollen, PT	Ortholog of AtAGP23, active during germination	[42]
		*AGP23* *(A. thaliana)*	Pollen, PT	Maintenance of PT growth	[43]
	Without GPI anchor	*AGP40* *(A. thaliana)*	Pollen	Formation of viable pollen	[43]
Chimeric AGPs: FLA	Fasciclin-like domain (FAS), GPI anchor, involved in adhesion and signaling data	*FLA3* (*A. thaliana*)	Microspores, developing pollen	Affects cellulose deposition, cell growth	[44]
		*CoFLA7* (*Camellia oleifera*)	PT	Enhances PT elongation	[45]
		*FLA14* *(A. thaliana)*	Pollen grain	Pollen development, intine formation, prevention of premature germination	[46]
		*FLA20* *(A. thaliana)*	Pollen grain	Participating in cell surface adhesion	[47]
		*MTR1* *(orthologist of AtFLA20)*	Pollen grain	Timely tapetum vacuolization, microspore formation / abnormal microspore morphology and male sterility	[48]
		*OsFLA1* (*O.sativa*)	Pollen grain	Exine development / shriveled pollen	[49]
Chimeric AGPs: ENODL	Nodulin-like domain, AG-glycomodule	*EN11–EN15* (*A. thaliana*)	Micropylar region of synergids, filiform apparatus	Required for PT reception, reception defects	[50,51]
Pistil-specific glycoproteins (non-classical AGPs)	Extensin-like or proline-rich, with AG-like glycans	*TTS* (*N. tabacum*)	Style transmitting tissue	Stimulates PT growth, chemoattraction, nutrition	[52,53]
		*PELPIII* (*N. tabacum*)	Style extracellular matrix	Species-specific PT growth inhibition (InInc)	[54,55]
		*120 K* (*N. tabacum*, *N. alata*)	Transmitting tissue	Required for S-specific rejection of incompatible pollen (binds S-RNases)	[56,57]
	Contains histidine domain	*NtPRP* (*N. tabacum*)	Pistil (transmitting tract)	Structurally similar to TTS, presumably involved in interspecific barriers	[58]
		*PRP1* *(P. hybrida*, *C. annuum*, *S. lycopersicum*, *S. tuberosum)*	Pistil (transmitting tract)	Structurally similar to TTS, associated with SI or InInc	[59,60]

The table summarizes the main AGP subfamilies. For each type, key structural features, representatives, and their known functions are indicated. Empty cells denote data not available in the reviewed literature.

## Data Availability

No new data were created or analyzed in this study. Data sharing is not applicable to this article.

## Abbreviations

The following abbreviations are used in this manuscript:

AGP/AGPs	Arabinogalactan protein(s)
AG peptide(s)	Arabinogalactan peptide(s)
FLA/FLAs	Fasciclin-like arabinogalactan protein(s)
GPI	Glycosylphosphatidylinositol
PG/PGs	Pollen grain(s)
PT/PTs	Pollen tube(s)
PAST domain	Proline, alanine, serine, and threonine domain
SI	Self-incompatibility
InInc	Interspecific incompatibility
S-RNase	S-Ribonuclease
TTS	Transmitting Tissue Specific
PELPIII	Pistil-specific extensin-like protein III
120 K	120-kDa glycoprotein
NtPRP	*Nicotiana tabacum* proline-rich protein
CRISPR/Cas9	Clustered Regularly Interspaced Short Palindromic Repeats/CRISPR-associated protein 9
PME4	Pectin methylesterase 4
